# Differentiation of Mouse Embryonic Stem Cells to Lympho-Hematopoietic Lineages*in Vitro*

**DOI:** 10.1155/1992/79518

**Published:** 1992

**Authors:** Una Chen

**Affiliations:** Basel Institute for Immunology Basel CH-4005 Switzerland

**Keywords:** ES cell differentiation, B lymphocyte, T lymphocyte, lymphoid vessel

## Abstract

Mouse embryonic stem (ES) cells can differentiate in culture to late stages of many cell lineages. have found culture conditions that are favorable for development *in vitro* of ES cells into hematopoietic cells at a stage equivalent to day 11-14 of fetal liver development. describe here: (1) the growth conditions necessary for maintenance of ES cells in an undifferentiated state, and the conditions that allow differentiation of cystic embryoid bodies that contain precursors of most hematopoietic cell lineages, including lymphoid cells; (2) the development of lymphoid vessels from ES fetuses*in vivo*; (3) the characterization of lymphoid, erythroid, megakaryoid, and myeloid cells from ES fetuses; and (4) the cloning of cell lines representing lymphoid, myeloid lineage cells from differentiated ES cells.


*Developmental Immunology*, 1992, Vol. 2, pp. 29–50
Reprints available directly from the publisher
Photocopying permitted by license only

© 1992 Harwood Academic Publishers GmbH
Printed in the United Kingdom

### Differentiation of Mouse Embryonic Stem Cells to Lympho-Hematopoietic Lineages *In Vitro*

UNA CHEN

*Basel Institute for Immunology, CH-4005 Basel, Switzerland*

Mouse embryonic stem (ES) cells can differentiate in culture to late stages of many cell lineages. I have found culture conditions that are favorable for development *in vitro* of ES cells into hematopoietic cells at a stage equivalent to day 11–14 of fetal liver development. I describe here: (1) the growth conditions necessary for maintenance of ES cells in an undifferentiated state, and the conditions that allow differentiation of cystic embryoid bodies that contain precursors of most hematopoietic cell lineages, including lymphoid cells; (2) the development of lymphoid vessels from ES fetuses *in vivo;* (3) the characterization of lymphoid, erythroid, megakaryoid, and myeloid cells from ES fetuses; and (4) the cloning of cell lines representing lymphoid, myeloid lineage cells from differentiated ES cells.

KEYWORDS: ES cell differentiation, B lymphocyte, T lymphocyte, lymphoid vessel.

### INTRODUCTION

Mouse embryonic stem cells (ES) derived from the inner cell mass (ICM) stage of embryogenesis retain their totipotency *in vivo* and *in vitro* (Gossler et al., 1986; Doetschman et al., 1987; Thompson et al., 1989; Koller and Smithies, 1989; Schwartzberg et al., 1989; Zijlstra et al., 1989; DeChiara et al., 1990; Koller et al., 1990). *In vitro,* ES cells differentiate into endoderm, ectoderm, and mesoderm (Martin and Evans, 1975; Martin, 1977; Robertson, 1987). The development *in vitro* of blood island or erythropoietic stem cells equivalent to the yolk sac stage (i.e., day 8–10 of embryogenesis) has been reported (Doetschman et al., 1985; Gossler et al., 1986; Robertson, 1987). Furthermore, erythroid and myeloid lineages containing megakaryocytes were also obtained by Cudennec and Johnson (1981) while studying the differentiation of teratocarcinoma cells *in vitro*. Recently, the mechanism of developmental regulation of the $\beta$-globin was studied with ES cells *in vitro* (Lindenbaum and Grosveld, 1990; Wiles and Keller, 1991; Schmitt et al., 1991).

The origin of the pluripotent stem cells and their lineage commitment to lymphocytes from mouse embryos are important issues in developmental biology that have recently received much attention. Stem cells from yolk sacs have been shown to give rise to erythrocytes, megakaryotic cells, and myeloid cells (Labastie et al., 1984). B lymphocytes can also be found after yolk sac stem cells are transferred into animals, but not in *in vitro* differentiation condition (Melchers, 1977; Paige et al., 1979; Melchers and Abramczuk, 1980; Chris Paige, personal communication). On the other hand, stem cells obtained from the fetal liver can give rise to erythroid, myeloid, and B-lymphoid cells *in vitro* (Owen et al., 1975; Melchers, 1977; Ogawa et al., 1988; Rolink et al., 1991). Differentiation of T-lymphoid cells requires a thymic microenvironment (see review by von Boehmer, 1990).

Pluripotent stem cells have been difficult to study because they are rare cells found in the heterogeneous cellular populations of bone marrow and fetal liver. Great progress has been made in obtaining subpopulations of bone marrow from adult and fetal liver that are highly enriched for pluripotent stem cells. Yet, very little is known about how stem cells commit to differentiation and how stem cells self-maintain. This lack of knowledge is at least partially due to the inability to maintain stem cells *in vitro.* Culture systems that would allow the growth of very primitive stem cells, such as mouse embryonic stem cells and the primitive hematopoietic stem cells, would be invaluable in addressing these issues.

The liquid culture system described in this study allows the development of pluripotent hematopoietic stem cells and all known hematopoietic lineages, including lymphoid (T and B) lineages from ES cells. Because this is an important step for studying lymphohematopoiesis *in vitro*, and because any deviation from the culture condition described in this study did not give rise to lymphocytes, I therefore describe *in detail* the differentiation conditions, as well as the growth conditions important for maintaining undifferentiated ES cells.

Differentiation *in vitro* can be followed under the microscope by observing the growing cells in three dimensions. First, they form simple embryoid bodies within clusters of cells and then cystic embryoid bodies with a complicated inner structure are developed. Finally, the embryoid bodies become "ES fetuses," which contain pulsating cardiac muscle, yolk sac blood islands, and, in addition, *novel* cup-shaped structures containing lymphoid cells. Further development of these ES fetuses was seen following introduction under the kidney capsule of young adult mice. From selected "ES fetuses," I use a variety of techniques to establish lymphoid and myeloid cell lines.

### RESULTS

### Differentiation of ES Cells to Simple, Then Cystic Embryoid Bodies, and the Selection of ES Fetuses Among Cystic Embryoid Bodies

Mouse ES cells derived from strains 129 (ES-D3) and C57B1/6 (ES-B6) grow two dimensionally on top of nondividing feeder cells, which are primary embryonic fibroblasts. ES cells were removed from feeder cells and allowed to differentiate in a liquid suspension culture. Within 24 hr, the cells aggregate and small round structures are formed. After 4–5 days, an outer layer of large endoderm cells is formed; some of them develop cavities. An inner layer of "ectodermal-like" cells is formed. These are *simple* embryoid bodies. From 8 to 30 days, there is a more complicated internal structure, which includes several easily recognizable mesodermal tissues such as blood cell clusters and cardiaclike tissue. These are *cystic* embryoid bodies (Martin and Evans, 1975; Cudennec and Nicolas, 1977; Doetschman et al., 1985; Gossler et al., 1986; Robertson, 1987). Depending on the origin of the ES cell line and the number of passages in culture, about 0.1–1% of the ES cells develop into *cystic* embryoid bodies. Under the liquid culture condition described in the Materials and Methods section, which allows a slow and free movement of embryoid bodies, I found that about 30–40% of the cystic embryoid bodies develop into "ES fetuses" (Fig. 1). The ES fetuses differentiated 10× more efficiently in the hydrophobic Heraeus tissue culture dish than the bacterial culture dish (see Materials and Methods section for source of dish). Fully formed cystic embryoid bodies, referred to as ES fetuses, are characterized by the presence of pulsating cardiac muscle, yolk sac blood islands, and, most importantly, the cup-shaped structures (large arrows in Fig. 1) containing small round 7–10-$\mu$m diameter lymphoid-like cells (small arrows in Fig. 1). They can be selected at various growth stages of differentiation from day 11 to 30.

### Characterization of ES Fetuses *In Vitro* and *In Vivo*

ES fetuses that were identified and selected under the phase contrast microscope, Figs. 2(A) and 2(B), were characterized histologically. As shown in Fig. 2(C), cardiac muscle cells and the cavity formed by the epithelium cells (cup-shape structure under the microscope) are visible. The clusters of cells inside the cavity shown in Fig. 2(D) are identified to be erythroid cells (arrow 5), myeloid cells (arrow 2 and arrow 4), and lymphoid cells (arrow 1).

ES fetuses were subsequently implanted under the kidney capsules of male Balb/c nude mice for 3 weeks, excised, and frozen. As shown in Figs. 2(E) to 2(I), immunophenotyping of the cryosections was performed. The sections were double stained for anti-H-$2K^b$ (conjugated to Texas Red, to demonstrate the class-I haplotype of ES origin) plus either anti-CD3 (conjugated to FITC) or anti-$\mu$ (conjugated to FITC). Both T cells and B cells were identified. T cells were identified in Figs. 2(F) and 2(G) by being positively stained with anti-CD3 alone (green color in Fig. 2(F), solid small arrow) or double stained with anti-CD3 plus anti-H-$2K^b$ (orange color in Fig. 2(G), solid small arrow). B cells were identified in Figs. 2(H) and 2(I), by positively stained with anti-$\mu$ alone

TABLE 1
Morphological Characterization of Lymphoid and Myeloid Cell Types Established from ES Cells

| Group | Methods of immortalization | Strain origin | Cell types[a] | | | | | | |
|---|---|---|---|---|---|---|---|---|---|
| | | | Lym[b] | Mo[b] | RBC[b] | Mega[b] | Mono[b] | Platelet | fat cells |
| 1 | A-MuLV | 129, C57B1/6 | + | + | + | + | + | + | + |
| 2 | J2 | 129 | + | + | + | + | – | – | – |
| 3 | RIM | 129, C57B1/6 | + | + | + | – | – | – | – |
| 4 | *myc+mil* | 129 | – | + | + | + | – | – | – |
| 5 | Growth factors (IL-3, IL-2+ConA) | 129, C57B1/6 | + | + | + | + | – | – | – |
| 6 | RIM+IL-3, +IL-2+ConA | 129, C57B1/6 | + | + | + | + | – | – | – |
| 7 | Growth factor IL-7 | 129 | + | + | + | + | + | – | – |

[a]Starting 2 weeks after retrovirus infection/lymphokine stimulation, the morphology of cell types was checked periodically for 6 months. The pattern of cell types was stable from about 4 weeks, if they were kept in the culture. Figure 3 shows representative individual hematopoietic cell types established from ES cells.
[b]Lym: lymphocytes; Mo: macrophages; Mono: monocytes; Mega: megakaryocytes; RBC: erythrocytes.

example, the *myc* and *mil* oncogenes (Group 4) produced cells mainly of the myeloid lineage. The cells established with growth factors (Groups 5 and 7) and J2 virus (Group 2) gave rise to both lymphoid and myeloid cells. Abelson (Group 1) and RIM viruses (Group 3) produced the broadest spectrum of cell types. The combination of RIM virus and growth factors (Group 6) established cell populations that differentiated into plasma cells, and mature T-cells, in adoptive transfer experiments (Chen et al., in preparation).

### Characterization of Cell Types by Morphology and *In Situ* Immunofluorescence

Cytocentrifuge preparations of each group were evaluated following staining with Giemsa. Table 1 summarizes the diversity of cell types found in each group after the establishment of stable cell populations, which required a period of 4 weeks to 6 months of culture. I started to examine the morphology of the cell types 2 weeks after "stimulation" (infection and/or growth factors) and found that the phenotypes were stable from 4 weeks onwards. Figure 3 shows the morphology of individual cells from one of these cultures (Group 6). Cells with morphology characteristic of monocytes (Fig 3, arrow 2) and macrophages (Fig. 3, arrow 3) were observed, and many of them were found to cluster around erythrocytes (Fig. 3, arrows 5 and 5'). Megakaryocytes (Fig. 3, arrow 4) were also found to be associated with these clusters. Lymphoidlike cells (Fig. 3, arrow 1) with little cytoplasm and dark-blue-staining nuclei were observed, either in clusters or interspersed between irregularly shaped, larger, light-blue-staining myeloid cells. Cells grown in the presence of cytokines and mitogens (Groups 5, 6, and 7) contained a significant number of mitotic figures, and many of them appeared to be irregular and contained multiple vacuoles, possibly due to spontaneous or virally induced transformation during long-term culture (i.e., up to 6 months). Although the

FIGURE 2. Characterization of "ES fetuses." Phase-contrast photomicrographs of one ES fetus. (A) Low magnification (5×) showing the whole body. C=cup-shape structure. H=heart. (B) Higher magnification (20×) focusing on a cup-shape structure containing a red dot of cells in the middle (arrow). (C) Azura A-Eosin B staining of an ES fetus in plastic embedding from (A) (low magnification, 5×). (D) Hematopoietic cells (magnification 100×) found in the cup-shape structure shown in (C): arrow 1= lymphocyte; arrow 2=monocyte; arrow 3=mitotic cell; arrow 4=megakaryocyte; and arrow 5=reticulocyte. (E) A Giemsa-stained frozen section of ES fetus under the kidney capsular (arrow), excised from Balb/c nude mice 3 weeks after implantation (magnification 3×). (F to I) Immunophenotyping of lymphocytes (solid small arrow) inside the lymphoid vessels (hollow large arrow) from mature ES fetuses as shown in (E) (magnification 40×). The sections were stained with (F) FITC-labeled anti-CD3 antibody (green color, solid small arrow) and (G) anti-H-2K$^b$ plus rat antimouse IgG-Texas Red (red color, hollow small arrow); (G) when double stained (CD3$^+$, H-2K$^{b+}$), the color was orange (solid small arrow). The sections were also stained with (H) anti-$\mu$ conjugated with FITC (green color, solid small arrow) and (I) anti-H-2K$^{b+}$ plus rat antimouse IgG Texas Red (red color, hollow small arrow); and (I) double stained (orange color, solid small arrow); (G and I) Note that some cells expressed only H-2K$^b$ surface antigen (red color, hollow small arrow). (See colour plate II at the back of this publication).

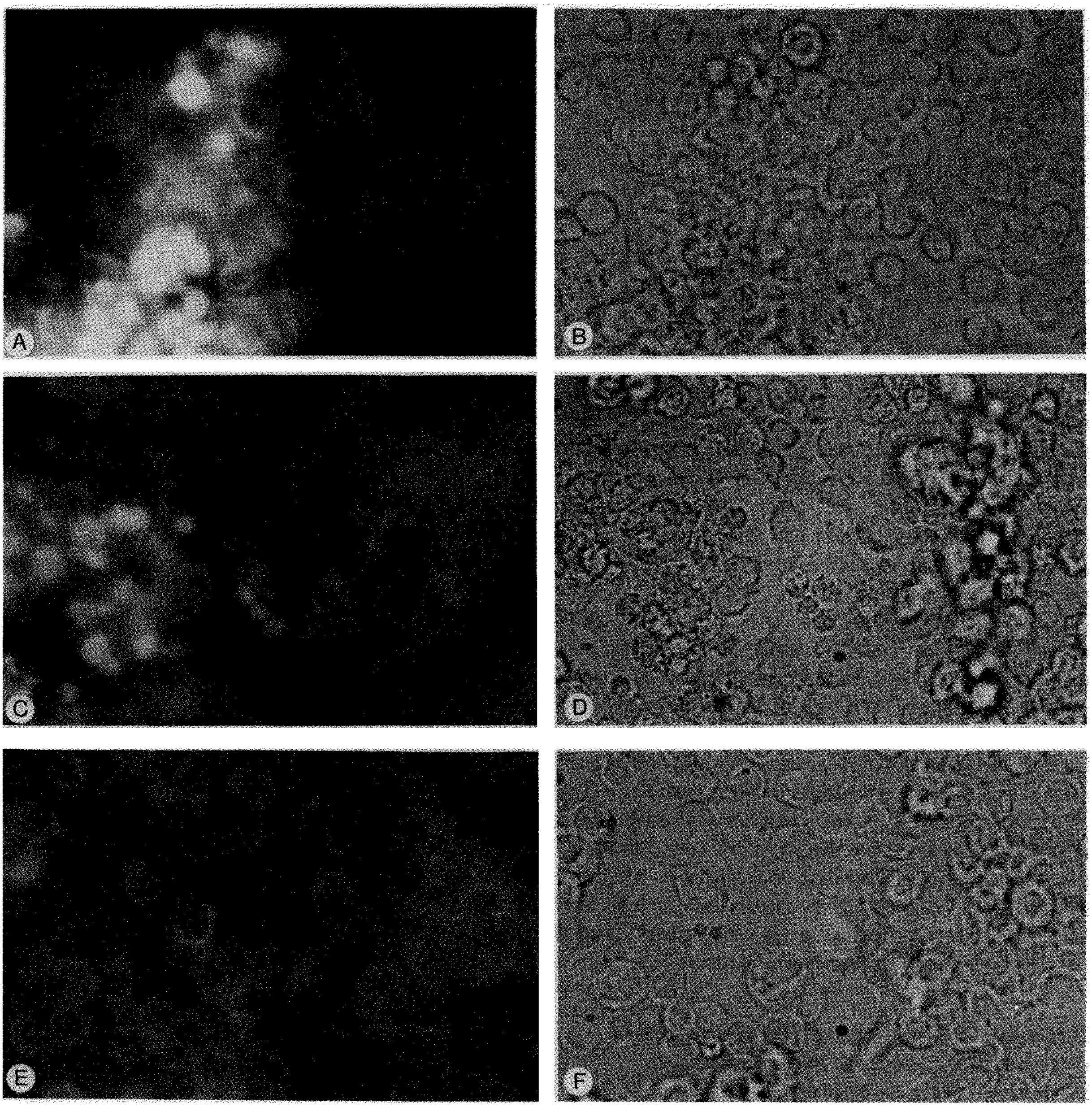

FIGURE 4. Differentiated lymphoid cells derived from ES cells identified by staining of lymphoid-cell-surface markers by indirect immunofluorescence microscopy. Photomicrographs of the cells from Group 6 (see Table 1). (A, C, and E) Immunofluorescent staining. (B, D, and F) Corresponding phase-contrast photomicrographs. (A) B-lymphoid cells identified by FITC-labeled sheep antimouse $\mu$-chain F(ab)'2 fragment. (C) T-lymphoid cells identified by anti-CD3 antibody (500-A2) and second antibody of FITC-labeled rabbit antihamster IgG F(ab)'2 fragment. (E) Second antibody alone (negative control). The staining was performed after six months of culture.

### Characterization of Surface Antigen Expression of Cell Populations by Flow Cytometry

As summarized in Table 2 and in one example of a histogram (Fig. 5), cells from each group were first analyzed for expression of B-cell markers such as surface Ig (sIg) and B220 antigens as well as the pre-B-cell marker identified by mAb G-5-2. A significant amount of sIg$^+$ cells was present in cells from different groups after retrovirus infection. Interestingly, cells from Group 7 did not express sIg. However, they possessed precursor B-cell markers such as G-5-2. The expression of B220 antigen did not correlate with sIg

TABLE 2
Percentage of Cells Expressing Positive Surface Markers in the ES-Derived Hematopoietic Cells as Analyzed by Immunofluoresence Labeling and Flow Cytometry

| Designation (group-cell population) | G-5-2 | sIg | B220 | CD4 | CD8 | CD3 | Thyl | Macl | SSEA | S7 |
|---|---|---|---|---|---|---|---|---|---|---|
| 1–5 | 3.77 | 17.92 | 1.42 | 0.79 | 0.67 | 10.36 | 0.35 | 35.59 | 3.18 | 24.02 |
| 2–41 | 12.40 | 16.45 | 0.85 | 1.31 | 1.83 | 18.26 | 1.77 | 74.33 | 3.49 | 19.86 |
| 3–3 | 2.80 | 5.30 | 6.18 | 0.0 | 0.0 | 4.8 | 1.0 | 11.0 | 0.50 | 41.70 |
| 5–47 | 44.86 | 31.78 | 35.90 | 47.05 | 0.00 | 22.51 | 36.94 | 29.09 | 0.49 | 39.36 |
| 6–44 | 13.66 | 16.67 | 21.23 | 0.00 | 0.00 | 10.39 | 57.57 | 32.47 | 1.10 | 2.15 |
| 7–45 | 6.08 | 0.71 | 20.04 | 9.97 | 0.78 | 0.52 | 17.88 | 8.48 | 1.55 | 4.43 |

expression. Also, Thyl antigen was not always present. The reason for this is not clear. It may suggest that it was an unusual phenomenon caused by the viral infection. In most groups, a signifciant number of cells expressed CD3 antigens, although most of them were $CD4^-$, $CD8^-$ cells. It is very interesting that Group 7 possessed $CD4^+$ single positive (i.e., $CD3^-$, $CD8^-$) cells. Whether these are the precursor cells resembling stem T cells found in the adult thymus (Wu et al., 1991) remains open. All groups contained cells of the myeloid lineages as defined by the mAb Mac-1. In all groups, most of the cells did not express SSEA-1 antigen, which appeared only at the blastocyst stage (Solter and Knowles, 1978), but retained various portions of embryonic antigen recognized by the mAb S7 (see what follows for a more detailed description of these antibodies). These data suggest that progenitor cells, precursor lymphoid cells, and some T and B cells exist in these mixed populations.

### Cloning and Characterization of Lymphoid, Myeloid, and Precursor Stem Cells from ES-Derived Hematopoietic Cells

One mixed population of hematopoietic cells established by using RIM virus infection (Group 3) was cloned on irradiated splenic feeder cells. The cloning frequency was 1 in 10 cells, because they were plated at 0.3 cells per well—37 clones out of 384 cultures containing growing cells. According to Poisson's distribution, about 90% of these positive cells could be derived from a single cell. These clones were divided into six subgroups according to three cell-surface-marker expressions (i.e., G-5-2, CD3, and $H\text{-}2K^b$). Some clones were characterized in detail by using a panel of mAbs and flow cytometry. As shown in Fig. 6, cells from primitive stem cells to lymphoid and myeloid lineages were established. All clones were grown in an ES differentiation medium (see Materials and Methods section) without cytokines. They have been in continuous culture for 1 year without alteration of growth behavior and phenotype.

All clones have lost the expression of SSEA-1 antigen, which was detected by mAb MC480 (Solter and Knowles, 1978). This antigen expresses on cells of the blastocyst stage of embryogenesis and is mostly lost when embryonic cells differentiate. Some clones have retained embryonic antigens detected by mAb S7. S7 has been described to detect pro-B cells (Hardy et al., 1989). We found that it detects a wide range of embryonic cells expressed from the blastocyst stage to the midsomite stage on most embryonic tissue (Chen et al., unpublished). Thus, I use this mAb to detect the stage of differentiation, the stage where the loss of antigens detected by S7 is roughly equivalent to the stage where increasing expression of H-2 class-I antigen is found.

As shown in Fig. 6(A), clone 19.1 is a T-cell clone because it expresses surface antigen of T-cell phenotype, that is, $CD3^+$, $\alpha b TCR^+$, $\gamma d TCR^-$, $CD4^-$, $CD8^-$. Clones 3.8 in Fig. 6(B) is as pre-B-cell clone; it expresses surface markers of precursor B-cell phenotype G52+, $B220^-$, $sIg^-$. Clone 3.2 in Fig. 6(C) is an immature myeloid cell clone; it expresses $Mac\text{-}1^+$ and $S7^+$ phenotype. Clone 3.12 in Fig. 6(D) is a possible natural killer (NK) cell or a precursor cell; it expresses NK1.1 surface marker. Other hematopoiesis markers tested are $Lyl^-$, $Ack^{\pm}$, and $Pgp1^+$ (histograms not shown). This clone killed sublethally irradiated adult mice (10 out of 10) 10–14 days upon intraperitoneal injection. Clone 3.25 in Fig. 6(E) is a

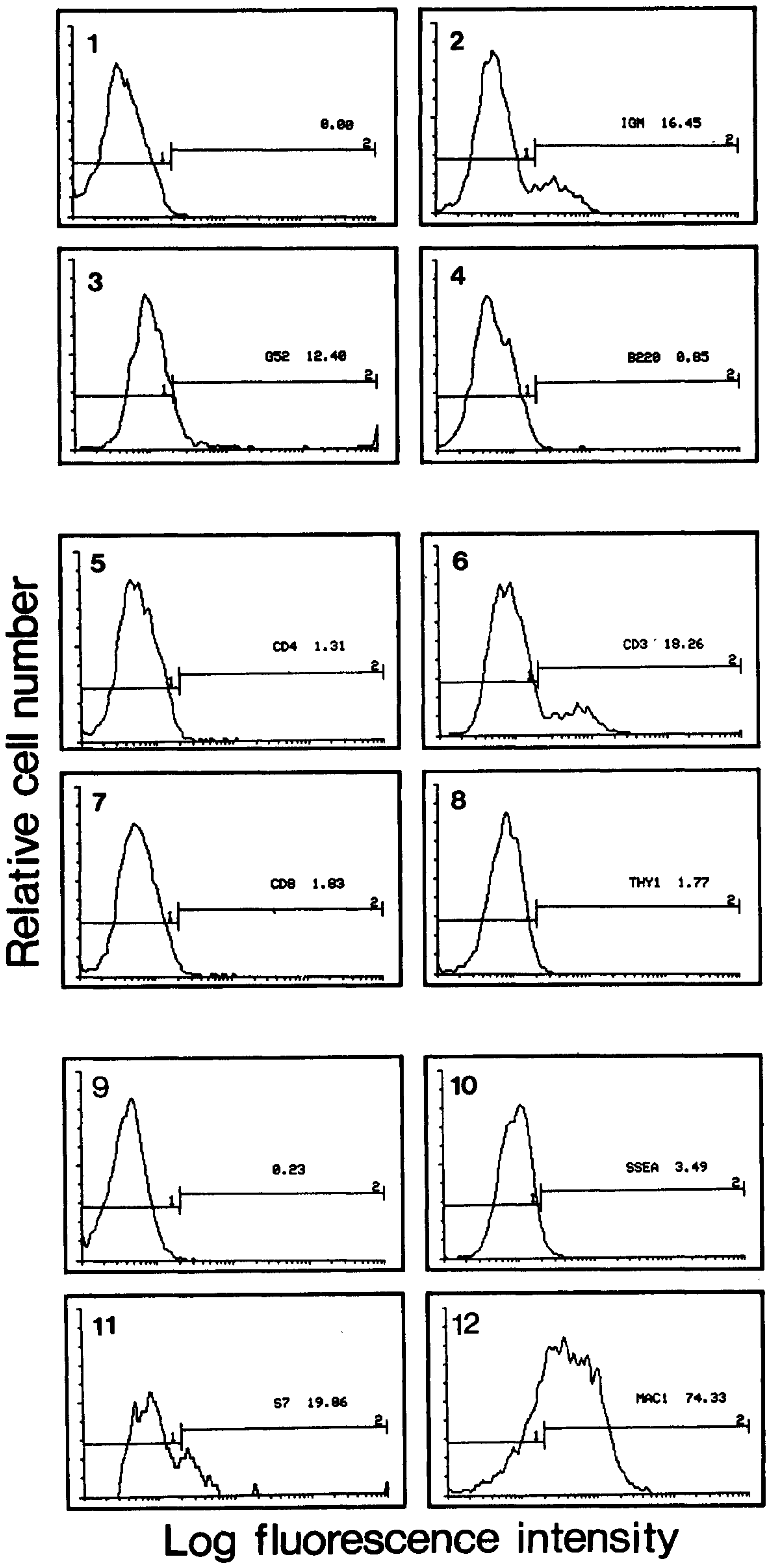

FIGURE 5. Flow cytometric analysis of lymphohematopoietic cells derived from ES cells (Group 2). The numbers indicate the percentage of positive cells of each individual staining antibody as indicated in each histogram. mAbs used are indicated in each panel and described in detail in the Materials and Methods section. In brief, 1: negative control; 2: FITC-labeled goat antimouse $\mu$-chain; 3: biotin-labeled G-5-2 and PE-labeled streptavidin; 4: FITC-labeled 14.8 (anti-B220); 5: PE-labelled GK1.5 (anti-CD4); 6: FITC-labeled 500-A2 (anti-CD3); 7: FITC-labeled 53-6-75 (anti-CD8); 8: FITC-labeled T-24 (anti-Thy1); 9: FITC-labeled mouse antirat IgG (second antibody control); 10: FITC-labeled MC480 (anti-SSEA-1); 11: rat IgG antimouse S7 and FITC-labeled mouse antirat IgG; 12: rat IgG antimouse M1/70 (anti-Mac-1) and FITC-labeled mouse antirat IgG. $1 \times 10^6$ cells per antibody staining was performed and a minimum of $1 \times 10^4$ cells were analyzed per sample. Cells were stained for cytometric analysis for 6 months of culture.

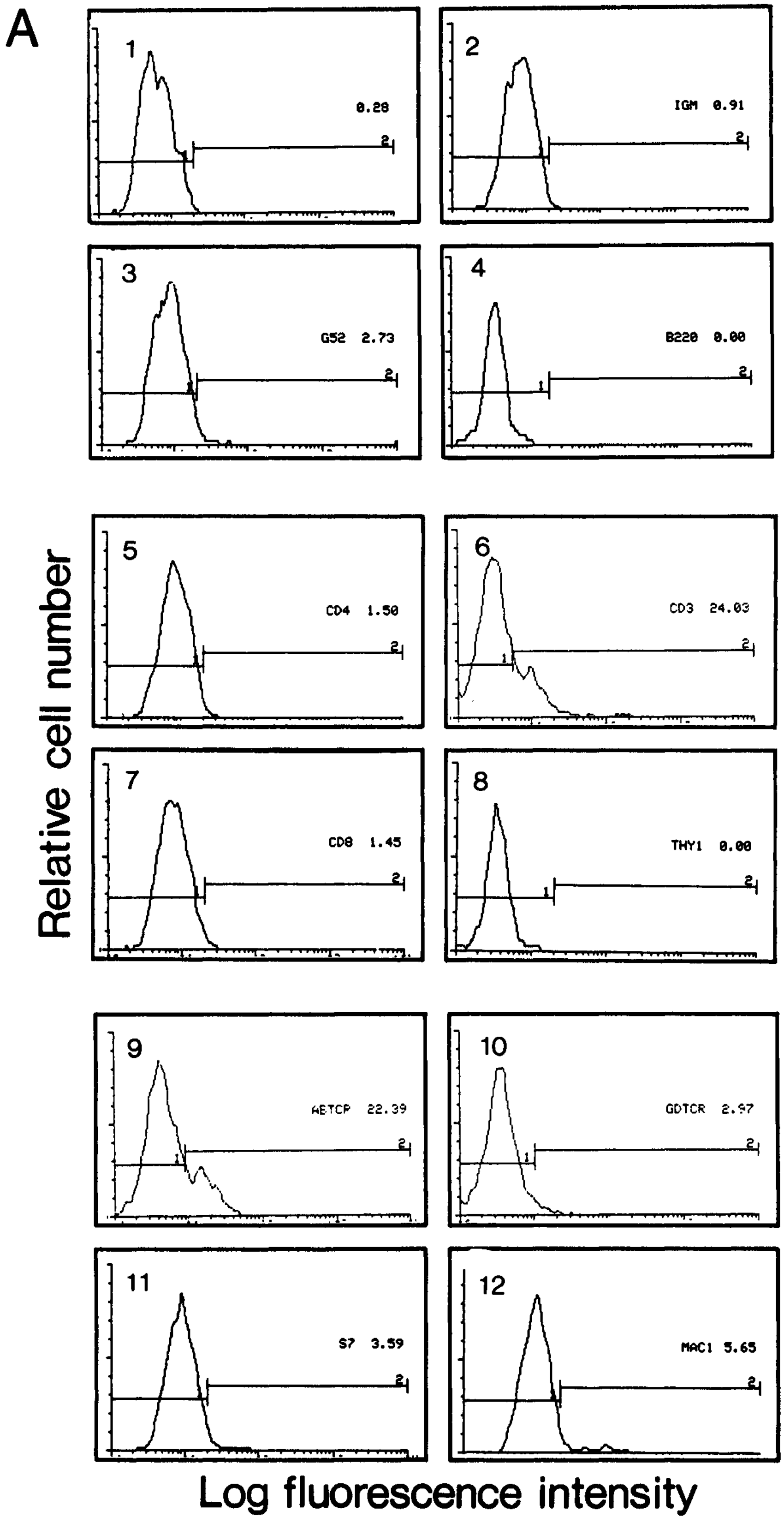

FIGURE 6. Flow cytometric analysis of lymphoid and myeloid cell lines derived from ES cells. (A to E) Numbers indicate the percentage of positive cells of each individual staining antibody as indicated in each histogram. mAbs used are indicated in each panel and described in detail in the Materials and Methods section. In brief, (A) 1: negative control; 2: FITC-labeled goat antimouse $\mu$-chain; 3: biotin-labeled G-5-2 and PE-labeled streptavidin; 4: FITC-labeled 14.8 (anti-B220); 5: PE-labeled GK1.5 (anti-CD4); 6: FITC-labeled 500-A2 (anti-CD3); 7: FITC-labeled 53-6-75 (anti-CD8); 8: FITC-labeled T-24 (anti-Thy1); 9: FITC-labeled H57-597 (anti-$\alpha b$TCR); 10: FITC-labeled GL3 (anti-$\gamma d$TCR); 11: rat IgG antimouse S7 and FITC-labeled mouse antirat IgG; 12: rat IgG antimouse M1/70 (anti-Mac-1) and FITC-labeled mouse antirat IgG. The second antibody control (FITC-labeled mouse antirat IgG) for 11 and 12 in A, C, D, and E were not included. B1 to 8 are the same as those indicated in A. 9: FITC-labeled mouse antirat IgG (second antibody control); 10: FITC-labeled MC480 (anti-SSEA-1); 11: rat IgG antimouse S7 and FITC-labeled mouse anti-rat IgG. 12: rat IgG antimouse M1/70 (anti-Mac-1) and FITC-labeled mouse antirat IgG. (C and E) Panels are the same as those indicated in B except 9, which is biotin-labeled B8-24-3 (anti-H-2K$^{b}$) and PE-labeled streptavidin. (D) Panels are the same as those indicated in B except 9, which is FITC-labeled pK136 (anti-NK1.1). 1x10$^{6}$ cells per antibody staining was performed and a minimum of 1x10$^{4}$ cells were analyzed per sample. ·Flow cytometric analysis was performed after the cell clones were in culture for 3 months.

FIGURE 6. Flow cytometric analysis of lymphoid and myeloid cell lines derived from ES cells. (A to E) Numbers indicate the percentage of positive cells of each individual staining antibody as indicated in each histogram. mAbs used are indicated in each panel and described in detail in the Materials and Methods section. In brief, (A) 1: negative control; 2: FITC-labeled goat antimouse $\mu$-chain; 3: biotin-labeled G-5-2 and PE-labeled streptavidin; 4: FITC-labeled 14.8 (anti-B220); 5: PE-labeled GK1.5 (anti-CD4); 6: FITC-labeled 500-A2 (anti-CD3); 7: FITC-labeled 53-6-75 (anti-CD8); 8: FITC-labeled T-24 (anti-Thy1); 9: FITC-labeled H57-597 (anti-$\alpha\beta$TCR); 10: FITC-labeled GL3 (anti-$\gamma\delta$TCR); 11: rat IgG antimouse S7 and FITC-labeled mouse antirat IgG; 12: rat IgG antimouse M1/70 (anti-Mac-1) and FITC-labeled mouse antirat IgG. The second antibody control (FITC-labeled mouse antirat IgG) for 11 and 12 in A, C, D, and E were not included. B1 to 8 are the same as those indicated in A. 9: FITC-labeled mouse antirat IgG (second antibody control); 10: FITC-labeled MC480 (anti-SSEA-1); 11: rat IgG antimouse S7 and FITC-labeled mouse anti-rat IgG. 12: rat IgG antimouse M1/70 (anti-Mac-1) and FITC-labeled mouse antirat IgG. (C and E) Panels are the same as those indicated in B except 9, which is biotin-labeled B8-24-3 (anti-H-2K$^b$) and PE-labeled streptavidin. (D) Panels are the same as those indicated in B except 9, which is FITC-labeled pK136 (anti-NK1.1). $1\times10^6$ cells per antibody staining was performed and a minimum of $1\times10^4$ cells were analyzed per sample. Flow cytometric analysis was performed after the cell clones were in culture for 3 months.

FIGURE 6. Flow cytometric analysis of lymphoid and myeloid cell lines derived from ES cells. (A to E) Numbers indicate the percentage of positive cells of each individual staining antibody as indicated in each histogram. mAbs used are indicated in each panel and described in detail in the Materials and Methods section. In brief, (A) 1: negative control; 2: FITC-labeled goat antimouse $\mu$-chain; 3: biotin-labeled G-5-2 and PE-labeled streptavidin; 4: FITC-labeled 14.8 (anti-B220); 5: PE-labeled GK1.5 (anti-CD4); 6: FITC-labeled 500-A2 (anti-CD3); 7: FITC-labeled 53-6-75 (anti-CD8); 8: FITC-labeled T-24 (anti-Thy1); 9: FITC-labeled H57-597 (anti-$\alpha\beta$TCR); 10: FITC-labeled GL3 (anti-$\gamma\delta$TCR); 11: rat IgG antimouse S7 and FITC-labeled mouse antirat IgG; 12: rat IgG antimouse M1/70 (anti-Mac-1) and FITC-labeled mouse antirat IgG. The second antibody control (FITC-labeled mouse antirat IgG) for 11 and 12 in A, C, D, and E were not included. B1 to 8 are the same as those indicated in A. 9: FITC-labeled mouse antirat IgG (second antibody control); 10: FITC-labeled MC480 (anti-SSEA-1); 11: rat IgG antimouse S7 and FITC-labeled mouse anti-rat IgG. 12: rat IgG antimouse M1/70 (anti-Mac-1) and FITC-labeled mouse antirat IgG. (C and E) Panels are the same as those indicated in B except 9, which is biotin-labeled B8-24-3 (anti-H-2K$^b$) and PE-labeled streptavidin. (D) Panels are the same as those indicated in B except 9, which is FITC-labeled pK136 (anti-NK1.1). $1\times10^6$ cells per antibody staining was performed and a minimum of $1\times10^4$ cells were analyzed per sample. Flow cytometric analysis was performed after the cell clones were in culture for 3 months.

FIGURE 6. Flow cytometric analysis of lymphoid and myeloid cell lines derived from ES cells. (A to E) Numbers indicate the percentage of positive cells of each individual staining antibody as indicated in each histogram. mAbs used are indicated in each panel and described in detail in the Materials and Methods section. In brief, (A) 1: negative control; 2: FITC-labeled goat antimouse $\mu$-chain; 3: biotin-labeled G-5-2 and PE-labeled streptavidin; 4: FITC-labeled 14.8 (anti-B220); 5: PE-labeled GK1.5 (anti-CD4); 6: FITC-labeled 500-A2 (anti-CD3); 7: FITC-labeled 53-6-75 (anti-CD8); 8: FITC-labeled T-24 (anti-Thy1); 9: FITC-labeled H57-597 (anti-$\alpha\beta$TCR); 10: FITC-labeled GL3 (anti-$\gamma\delta$TCR); 11: rat IgG antimouse S7 and FITC-labeled mouse antirat IgG; 12: rat IgG antimouse M1/70 (anti-Mac-1) and FITC-labeled mouse antirat IgG. The second antibody control (FITC-labeled mouse antirat IgG) for 11 and 12 in A, C, D, and E were not included. B1 to 8 are the same as those indicated in A. 9: FITC-labeled mouse antirat IgG (second antibody control); 10: FITC-labeled MC480 (anti-SSEA-1); 11: rat IgG antimouse S7 and FITC-labeled mouse anti-rat IgG. 12: rat IgG antimouse M1/70 (anti-Mac-1) and FITC-labeled mouse antirat IgG. (C and E) Panels are the same as those indicated in B except 9, which is biotin-labeled B8-24-3 (anti-H-2K$^b$) and PE-labeled streptavidin. (D) Panels are the same as those indicated in B except 9, which is FITC-labeled pK136 (anti-NK1.1). 1x$10^6$ cells per antibody staining was performed and a minimum of 1x$10^4$ cells were analyzed per sample. Flow cytometric analysis was performed after the cell clones were in culture for 3 months.

FIGURE 6. Flow cytometric analysis of lymphoid and myeloid cell lines derived from ES cells. (A to E) Numbers indicate the percentage of positive cells of each individual staining antibody as indicated in each histogram. mAbs used are indicated in each panel and described in detail in the Materials and Methods section. In brief, (A) 1: negative control; 2: FITC-labeled goat antimouse $\mu$-chain; 3: biotin-labeled G-5-2 and PE-labeled streptavidin; 4: FITC-labeled 14.8 (anti-B220); 5: PE-labeled GK1.5 (anti-CD4); 6: FITC-labeled 500-A2 (anti-CD3); 7: FITC-labeled 53-6-75 (anti-CD8); 8: FITC-labeled T-24 (anti-Thy1); 9: FITC-labeled H57-597 (anti-$\alpha\beta$TCR); 10: FITC-labeled GL3 (anti-$\gamma\delta$TCR); 11: rat IgG antimouse S7 and FITC-labeled mouse antirat IgG; 12: rat IgG antimouse M1/70 (anti-Mac-1) and FITC-labeled mouse antirat IgG. The second antibody control (FITC-labeled mouse antirat IgG) for 11 and 12 in A, C, D, and E were not included. B1 to 8 are the same as those indicated in A. 9: FITC-labeled mouse antirat IgG (second antibody control); 10: FITC-labeled MC480 (anti-SSEA-1); 11: rat IgG antimouse S7 and FITC-labeled mouse anti-rat IgG. 12: rat IgG antimouse M1/70 (anti-Mac-1) and FITC-labeled mouse antirat IgG. (C and E) Panels are the same as those indicated in B except 9, which is biotin-labeled B8-24-3 (anti-H-2K$^{b}$) and PE-labeled streptavidin. (D) Panels are the same as those indicated in B except 9, which is FITC-labeled pK136 (anti-NK1.1). $1\times10^{6}$ cells per antibody staining was performed and a minimum of $1\times10^{4}$ cells were analyzed per sample. . Flow cytometric analysis was performed after the cell clones were in culture for 3 months.

precursor stem cell; it has lost both SSEA-1 antigen and the S7 embryonic antigen, however, H-2K$^b$ class-I antigen is highly expressed (midsomite stage; Ozato et al., 1985) and this clone is negative for all other markers used in this study. The data suggest that using a retrovirus (such as KIM) to infect ES fetuses, it is possible to obtain mixed populations of hematopoietic cells. It is also possible to establish cell lines of lymphoid and myeloid lineages at different stages of development.

### The Hematopoietic Cells Arose from ES Cells, Not from Feeder Cells

Extreme care was taken to ensure that hematopoietic cells were established from ES fetuses, not from feeder cells. Primary embryonic feeder cells were derived from fibroblasts of nonlymphoid tissues. All the feeder cells were subjected to mitomycin C treatment or irradiation to render them incapable of dividing. The nondividing feeder cells perished after 1–2 passages (4–5 days) in the culture, whereas the differentiation of ES fetuses requires 11–30 days. In control experiments, feeder cells were grown under similar conditions but without ES cells; nothing remotely resembling embryoid bodies or ES fetuses was found. Although the feeder cells differed from the ES cells in H-2 haplotypes, undifferentiated ES cell lines do not express H-2 class-I antigens, Figs. 7(A) and (B). Low levels of H-2 class-I antigens are expressed at the midsomite stage (day 9), and the level of expression remains low until day 13 (Ozato et al., 1985). However, ES-cell-derived hematopoietic cells express H-2K$^b$, Fig. 7(C), as do cells of strain 129 from which the ES cells were derived; the feeder cells do not express this H-2K$^b$ haplotype, Fig. 7(D). In quantitative immunofluoresence measurements by flow cytometry, about half of the cells were H-2K$^b$ positive, Fig. 7(E). These cells do not express H-2D$^d$, which corresponds to the haplotype of the feeder cells, Fig. 7(F). ES cells and feeder cells must be trypsinized to obtain single cell suspensions, and this procedure destroys the surface antigens; hence, flow cytometric analysis of ES cells and feeder cells was not feasible. From these results with H-2 typing, I conclude that the hematopoietic cells cannot have arisen from the feeder cells.

### DISCUSSION

ES cells are cell lines cloned from the inner cell mass (ICM) of blastocysts. In this paper, I have described an embryonic organ culture system in which these totipotent cells differentiate into ES fetuses that contain various hematopoietic cells including lymphoid cells. Upon *in vivo* implantation, they develop into more mature lymphoid tissues. I have also decribed the establishment of cell lines by using retroviruses. Mixed populations of myeloid, erythroid, megakaryoid, and lymphoid cells were identified and clones of several cell types were established. It is intriguing that it is possible to infect "ES fetuses" with retroviruses containing oncogenes. Infection seemed to increase the cell mass and life-span of ES fetuses up to 6 months in culture, and the cells obtained from these ES fetuses contained lineages of hematopoietic cells. This system has many advantages for studying developmental immunology.

There are elements described in detail in the Materials and Methods section that are known to be essential for lymphohematopoietic stem cells to differentiate. The most important element is the "healthy" status of "undifferentiated" ES cells. LIF alone does not support any of the ES cell lines used in this study. The STO cell line, either alone or together with LIF, is not as supportive as the primary embryonic fibroblasts (data not shown). The periodical examination of the undifferentiated stage of ES cells by the criteria described is critical. Differentiation is found if the embryoid bodies are allowed to move freely, detached from a fixed environment (such as hydrophilic plastic surface of a regular tissue culture dish). The use of hydrophobic Heraeus tissue culture petri dishes seems to particularly favor the growth of ES fetuses. The daily feeding with cold medium containing high concentrations of good FCS also stimulates the growth. The feeding not only provides the nutrients but also the gentle stirring caused by the change of medium.

The development of ES fetuses from undifferentiated ES cells is not synchronous. Embryoid bodies grown in the same petri dish develop at various rates. It is important to select the ES fetuses from the original "master" dish at a critical time when all three of the following structures are present: blood islands (red in color),

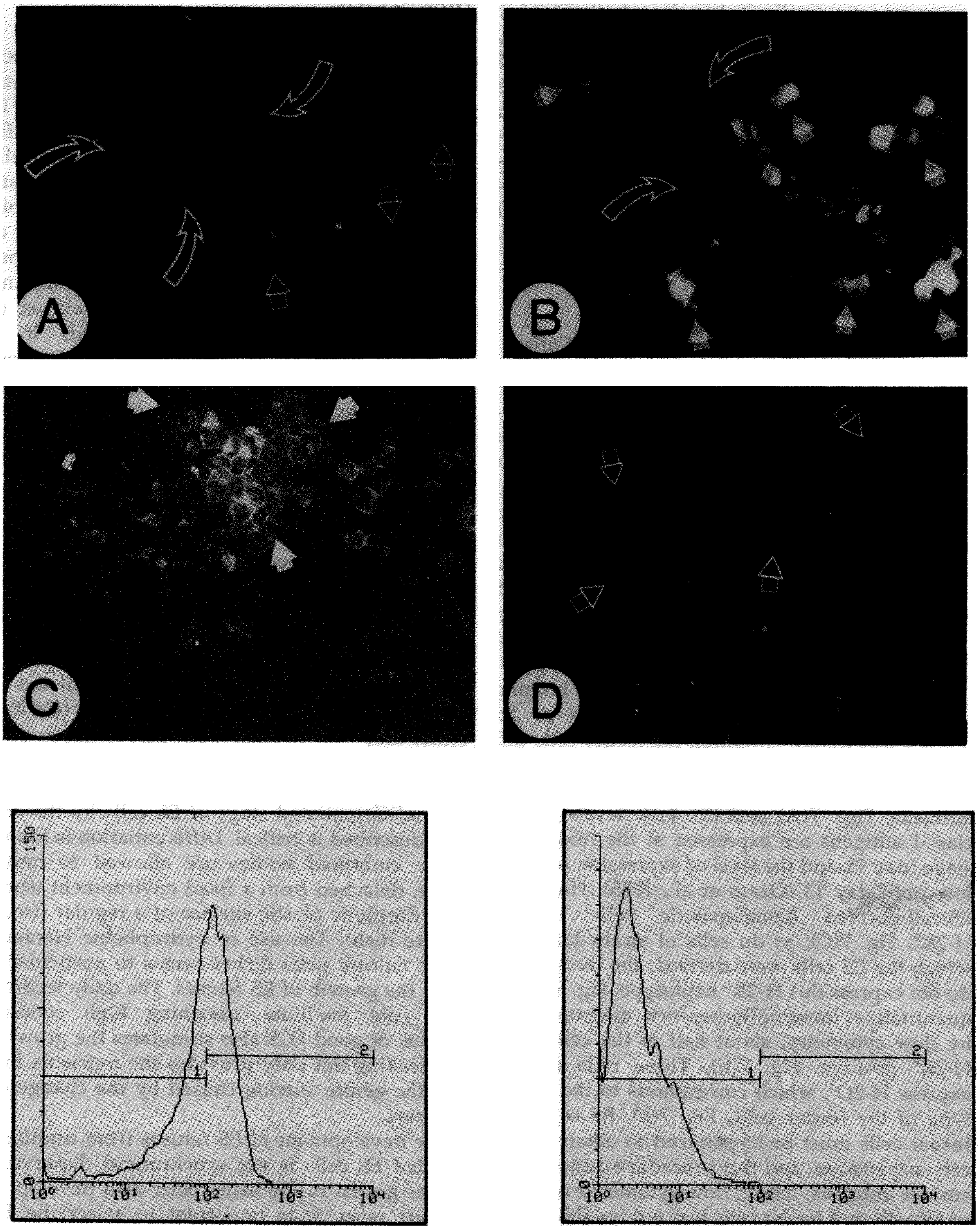

E

F

pulsating cardiac cells, and cup-shaped structure. It is especially important to look for the cup-shaped feature, which contains lymphoid cells. These selected ES fetuses are then placed in a new petri dish in groups of 5–10 and cultured further for infection with viruses and/or addition of interleukins. Prolonged culture in the master dish does not result in further differentiation of the embryoid bodies. Because no exogenous stimulating factors were included in the daily feeding medium, it was likely that the growth and differentiation seen were the result of factors derived from cells inside the embryoid bodies. It appeared that the selected ES fetuses contain "tissue" or its precursors important for lymphoid lineage development. Implantation of ES fetuses into adult animals gives rise to many tissues. Among them, lymphoid vessels containing positive surface markers of T and B cells were identifiable (this study, Fig. 2, and Chen et al., in preparation).

Although Moloney leukemia virus long terminal repetitive sequences (LTR) are inactive in the undifferentiated F9 teratocarcinoma (Gorman et al., 1985) and in ES cells (U. Chen, unpublished), the receptors for retrovirus must be generated during differentiation. I have immortalized cell lines by using retroviruses. Abelson viruses are shown to immortalize precursor B cells and many other stem cells. RIM viruses are known to immortalize plasma cells and were tumorgenic when they were injected intraperitoneally into pristane primed adult mice (Clynes et al., 1988), but this is the first indication that RIM may be able to immortalize hematopoietic cells. Preliminary data indicate that the vector was stably integrated into the cell lines (U. Chen et al., unpublished data). The establishment of stable clones of uncommitted stem cells, T cells, pre-B cells, myeloid cells, as well as NK cells, indicates that lymphoid and myeloid cells exist in the retrovirus-infected hematopoietic cell population derived from ES fetuses.

It is possible to obtain beating cardiac muscle, blood islands, and lymphoid-cell positive clusters, the so-called "ES fetuses," from ES lines derived from several different mouse strains. The ES-D3 cell line has been shown to be germ-line transmittable (Gossler et al., 1986; Doetschman et al., 1987). Although the ES-B6 cell line does not contribute to the germ line *in vivo*, I have obtained ES fetuses from this line, established hematopoietic cell lines, and found lymphocytes. Thus, the ability of a line to differentiate *in vitro* is not a predictor of its ability to form chimeras *in vivo*.

I have demonstrated that a variety of hematopoietic cells could be developed in this organ culture. It takes only 2 to 3 weeks for undifferentiated ES cells to progress to the fetal liver equivalent stage. ES fetuses developed in this culture contain pre-B cells capable of responding to bacterial lipopolysaccharide stimulation (U. Chen and F. Melchers, unpublished). A significant amount of these precursor B cells possess G-5-2 surface markers and these mitogen-responding pre-B cells are normally found between day 16–19 of fetal liver in mouse embryos (Melchers, 1977; Rolink et al., 1991; F. Melchers, personal communication).

The IL-2 and IL-7 responsive lymphocytes appear, at the earliest, at day 12–13 during embryogenesis (B. Imhof, personal communication). Adult thymus provides an environment for precursor cells to mature into intrathymic precursor cells that express mature T-cell marker CD4 (Wu et al., 1991). Fetal thymus appears earliest at day 10 of mouse embryogenesis and colonization by precursor stem cells is 1 day later, and parallels the development of fetal liver. The findings of IL-2- and IL-7-responsive lymphocytes and single $CD4^+$ cells from ES fetuses and the establishment of the $CD3^+$ $\alpha\beta TCR^+$, $CD4^-$ $CD8^-$ T-cell clone suggest that either ES fetuses provide thymus-equivalent environment or thymic environment is not neces-

FIGURE 7. Identification of H-2 haplotype of ES cells, feeder cells, and hematopoietic cells. Clusters of ES cells were derived from the inner cell mass of blastocysts originated from mouse strain 129 (H-$2K^b$) (hollow large arrows in A and B). The surrounding feeder cells were fibroblasts derived from the Balb/c origin (H-$2D^d$) (hollow small arrows in A and solid small arrows in B). Cells in A were treated with (A) fluorescence-labeled anti-H-$2K^b$ and (B) anti-H-$2D^d$ antibodies. Hematopoietic cells established from ES fetuses were treated with (C) fluoresence-labeled anti-H-$2K^b$ (solid small arrows) and (D) anti-H-$2D^d$ (hollow small arrows) antibodies. These cells were also subjected to quantitative immunofluorescent analysis by flow cytometry. (E) The histogram of the hematopoietic cells stained with anti-H-$2K^b$ antibody. (F) The histogram of these cells stained with anti-H-$2D^d$ antibody. (E and F) The x-axis indicates the log fluorescence intensity and the y-axis indicates the relative cell number.

sary for the maturation of these cells. The culture conditions described in this paper seem to be favorable for the development of lymphocytes equivalent to these stages.

Genetic manipulation of mouse ES cells (Thompson et al., 1989; Mansour et al., 1988; Koller and Smithies, 1989) and germ-line transmission of a specific defect (Schwartzberg et al., 1989; Thompson et al., 1989; Zijlstra et al., 1989; DeChiara et al., 1990; Koller et al., 1990) have provided a very powerful tool for studying gene function. However, if the knockout of some genes may affect normal embryonic development and/or the survival of the fetus, germ-line transmission cannot be achieved. The *in vitro* system described here may be able to provide an alternate way for studying the function of these genes.

### MATERIALS AND METHODS

### Maintenance and Identification of Undifferentiated ES Cell Lines

Mouse ES cells derived from strain 129 (CCE ES cells, 129/SV/Ev; Robertson, 1987; Schwartzberg et al., 1989) (ES-D3, 129/Sv, XY karyotype; Gossler et al., 1986) and C57B1/6 (ES-B6; Kemler, unpublished) were kindly provided by Elizabeth Robertson (Columbia University, NY) and Rolf Kemler (MPI for Immunbiology, Freiburg, FRG).

ES cells were kept in a humidified incubator (37 °C, 10% $CO_2$) and fed with DMEM (Gibco, Grand Island, NY, Cat. No. 041-01965M), supplemented with 4.5 g/ml glucose, 1 mM sodium pyruvate, 20 mM L-glutamine, penicillin (100 units/ml) (Gibco), streptomycin (100 units/ml) (Gibco), and 15% heat-inactivated (56 °C, 30 min) preselected fetal calf serum (FCS; Seromed, Biochrom KG, Berlin, FRG, Lot No. 2L03, or Boehringer, Mannheim, FRG, Lot No. 60039202). The FCS were selected following two criteria: (a) they give a high plating efficiency of ES cells at the undifferentiated stage (see Robertson, 1987, p.74, for detailed procedure). The screening of FCS requires a positive control lot of FCS for comparison, and (b) they are supportive in the splenic lymphoid cell culture system of Mishell and Dutton (1967) and are classified as "good FCS" by the Mishell's nomenclature (see Shiigi and Mishell, 1975; Mishell et al., 1978).

ES cells were kept undifferentiated (criteria are described in the Results section) in 60×15-mm tissue culture dishes (Falcon 3002, Becton Dickinson, Mountain View, CA) with nondividing feeder cells (see what follows). ES cells were used for less than 20 passages. ES cells, feeder cells, and all reagents are mycoplasma-free. It is necessary to maintain the cultures free of mycoplasma; no ES fetuses were formed whenever there was contamination.

Undifferentiated ES cells were identified in this study by (a) morphological appearance, (b) serology, and (c) ability to differentiate to hematopoietic lineages *in vitro*. Morphological characterization of ES cells by the criteria of Robertson (1987) was performed under the phase-contrast microscope. Morphologically, ES cell clusters resemble "pancakes" or "islands," and the edges of the individual cells are not clearly distinguishable. Once the cells begin to grow three dimensionally, and the edges of the clusters begin to be distinct, the culture has already moved toward "chaotic" differentiation. Undifferentiated ES cells express a stage-specific embryonic antigen (SSEA-1), which can be recognized by a monoclonal antibody, MC-480 (Solter and Knowles, 1978). When the cells start to differentiate, the expression of SSEA-1 is lost. Immunotyping provides a very sensitive, reliable, simple, and quick way to identify differentiated ES cells before the apparent morphological disorder.

### Feeder Cells for Maintenance of Undifferentiated ES Cell Lines

Primary embryonic fibroblasts were used to maintain ES cell lines at the totipotent, undifferentiated stage. Embryonic fibroblasts were prepared from day 13–14 mouse embryos as described by Gossler et al. (1986) and Rolf Kemler (Max Planck Institute for Immunobiology, Freiburg, FRG, personal communication). Head, thymus, fetal liver, gut, and hard tissues were removed. The remaining tissues were minced into small pieces with a pair of scissors, passed through a fine sieve, and digested for 30 min in Trypsin-EDTA (1×) (Gibco). Cells from 10–15 embryos were isolated and seeded in the medium described before and grown in one 150×15-mm tissue culture dish (Falcon 1058). After overnight culture, the nonadherent cells were removed. Fibroblasts were established after three to four passages. They were either used immediately or frozen in aliquots in liquid nitrogen tanks. All feeder cells were rendered incapable of division, either by treating with mitomycin C (Sigma, St. Louis, MO, 10 $\mu$g/ml) for a minimum of 2 hr (Martin and Evans, 1975) or irradiation (3,000 rad). LIF alone does not support the ES cells described in this study at the undifferentiated stage. The STO cell line, either alone or together with LIF, is not as supportive as the primary embryonic fibroblasts.

### *In Vitro* Differentiation of ES Cells to Simple, then Cystic Embryoid Bodies, and the Selection of ES Fetuses

A modification of published protocols (Gossler et al., 1986; Robertson, 1987) was used. Undifferentiated ES cells were removed from feeder cells and cultured in suspension using ES differentiation medium (the same medium as specified before with $10^{-4}$ M 2-mercaptoethanol). Undifferentiated ES cells were detached from the plate using trypsin-EDTA solution (Gibco), 1–2x$10^5$ cells were transferred to a 60x15-mm hydrophobic tissue culture dish (Heraeus, Heusenstamm, FRG) or if not available, to a 100x15-mm bacterial culture dish (Greine Nuringer, FRG). The small clusters of cell aggregates were grown in suspension at 37 °C in a humidified incubator with air and 5% $CO_2$ (and not 10% $CO_2$) for 30 days with daily change of medium (temperature of medium must be 4 °C). The clusters of cells expand three dimensionally to form simple embryonic bodies. Later on, complicated structures develop inside the bodies, which then become cystic embryonic bodies. From day 11 to 30, cultures were examined, and among cystic embryoid bodies containing complex embryonic structures, ES fetuses were selected by the following criteria: the presence of beating heart muscle cells, visceral yolk saclike blood islands, and small lymphoid clusters contained within a cup-shaped structure. Five to ten ES fetuses were pooled and transferred to another 60x15-mm tissue culture dish.

### *In Vitro* and *In Vivo* Characterization of ES Fetuses

ES fetuses were fixed in 4% formaldehyde in PBS, dehydrated, and plastic embedded by standard technique and cut to a thickness of 2 $\mu$m, and staining of the sections with Azura A and Eosin B was performed. ES fetuses were implanted under the kidney capsules of Balb/c Nu/Nu male mice (Jackson Lab., Maine) of 6 weeks of age. Three weeks later, mice were sacrificed and the mature ES fetuses together with the kidney were removed and frozen. Frozen sections were cut to a thickness of 5 $\mu$m on cryostat (Leitz, Wetzlan), fixed with acetone, rehydrated in PBS containing 1% bovine serum albumin, and immunophenotyping was performed with FITC-labeled goat antimouse $\mu$-chain antibody (Southern Biotechnology, Birmingham, AL), FITC-labeled anti-CD3 antibody (Havran et al., 1987), anti-H-2K$^b$ class-I antigen (Köhler et al., 1981), and Texas Red-labeled rat antimouse IgG antibodies (Jackson Lab., West Grove, PA). Immunofluorescence was then evaluated using a microscope as before.

### Development of Hematopoietic Cell Populations from ES Fetuses

Cystic embryoid bodies, selected as early as day 11 when blood islands started to appear up to 30 days when the blood islands turned brown, were immortalized by one of the following methods:

**Method 1.** Individual ES fetuses in separate dishes were infected with one of the following retroviruses: (i) A-MuLV (v-*abl;* Rosenberg et al., 1975; Rosenberg and Baltimore, 1976; gift of Fritz Melchers), (ii) J2 (v-*raf/mil*+*myc;* Troppmair et al., 1989; gift of Ulf Rapp), (iii) RIM (Ig-*myc*+v-Ha-*ras;* Clynes et al., 1988; gift of Ken Marcu), or (iv) *myc*+*mil* virus (v-*myc*+v-*mil;* Righi et al., 1989; gift of Ron-hwa Lin). Virus titers were $10^5$–$10^6$ Pfu/ml, and the infection was performed overnight in the presence of polybrene (10 $\mu$g/ml; Sigma). After infection, ES fetuses continued to grow for up to 6 months in culture with regular feeding until they became 0.5–1 cm giant bodies. Cells then detached from the bodies spontaneously.

**Method 2.** Individual ES fetuses were disrupted mechanically to release single cells or clusters of cells, which were seeded in separate dishes at $10^4$–$10^5$ cells/dish and either (a) infected with retroviruses at the 1 ml/dish as before and fed regularly with ES differentiation medium or (b) fed with ES differentiation medium containing growth factors and/or the mitogen, concanavalin A (ConA; Pharmacia, Uppsala; 50 $\mu$g/ml final concentration). The growth factors included recombinant interleukins-1 to -5 (30 U/ml final concentration, Karasuyama and Melchers, 1988; gifts of Fritz Melchers), which were supernatants from IL-producing transfectants, or erythropoietin (1 U/ml final concentration; Sigma). Recombinant IL-7 (100 to 200 U/ml final concentration) was a gift from Fritz Melchers. The IL-7 was produced by a cell line transfected with the mouse IL-7 gene (kindly provided by Shin-Ichi Nishikawa).

### Characterization of Hematopoietic Cell Populations Derived from ES Fetuses

**Morphology.** Cells were cytocentrifuged onto glass slides, methanol fixed and, May-Grünwald-Giemsa stained. The preparations were then cover slipped and evaluated using a Zeiss Axioscope 20 light microscope.

**Immunofluoresence Analysis.** Hematopoietic cells were allowed to grow for 2–3 days on sterile cover slips. Subsequently, the preparations were washed in PBS, immersed in acetone for 30 seconds, air dried, and labeled with antibodies. The antibodies used are FITC-labeled 500-A2, which recognizes CD3 antigen on T lymphoid cells (Havran et al., 1987); FITC-labeled rabbit antihamster IgG F(ab)'2 fragment and FITC-labeled goat anti-$\mu$ antibodies (Southern Biotechnology, Birmingham, AL); FITC-labeled sheep antimouse Ig (heavy and light chains) F(ab)'2 fragments (Silenus, Hawthorotreet, Australia); 15.5.5, which recognizes H-$2D^d$ class-I molecules of all cells of the H-$2^d$ haplotype (Ozato et al., 1980); and B8-24-3, which recognizes H-$2K^b$ class-I molecules on all cells of the H-$2^b$ haplotype (Köhler et al., 1981). Immunofluoresence was then evaluated under UV illumination on a Zeiss Axioscope 20 using filters for excitation of FITC and Texas Red. Besides the antibodies mentioned before, the following antibodies were used for flow cytometric analysis: T-24, which recognizes Thy-1 antigen (Dennert et al., 1980); GK1.5, which recognizes CD4 molecules on T cells (Wilde et al., 1983); 53-6-75, which recongizes CD8 molecules on T cells (Ledbetter and Herzenberg, 1979); H57-597, which recognizes all $\alpha b$ T-cell receptors (Kubo et al., 1989); GL3 which recognizes $\gamma d$ T-cell receptor (Goodman and LeFrancois, 1989); PK136, which recognizes NK1.1 antigen on natural killer cells (Koo and Peppard, 1984); G-5-2, which recognizes GP-76 on Pre-B and plasma cells (Strasser, 1988); 14.8, which recognizes B-220 molecules on B cells (Coffman and Weissman, 1981; Kincade et al., 1981); M1/70, which recognizes Mac-1 antigen on macrophages (Springer et al., 1979); S7, which recognizes most of the embryonic cells including pro-B cells (Hardy et al., 1989; Chen et al., unpublished) and FITC-labeled mouse antirat IgG (Jackson Lab., West Grove, PA). All antibodies were titrated and specificity was characterized using mouse splenic cells and thymocytes (data not shown). Flow cytometry was carried out with a FACS Analyzer (Becton Dickinson, Mountain View, CA). Fluorescence histograms were generated with logarithmic amplification of fluoresence emitted by a minimum of $1 \times 10^4$ single viable cells.

### Cloning and Characterization of Lymphoid and Myeloid Lineages from ES-Derived Hematopoietic Cells

A mixed population of cells from RIM virus-infected ES fetuses was cloned on feeder cells, which were 3,000-rad $\gamma$-irradiated splenic cells from Balb/c mice. Cloning of cell lines was performed by culturing cells at 0.3 cells/well in 96-well flat-bottomed microculture plates in the ES-differentiation medium as described before. After 7 days, the plates were examined for cultures showing positive growth of clones of cells under the microscope. Clones of cells were then transferred to 24-well culture plates and then to a tissue culture flask and were fed regularly. No feeder cells were required after the cloning. The cell-surface-marker expression of cell lines was examined using immunofluoresence staining of antibodies and flow cytometric analysis as described before.

### ACKNOWLEDGMENTS

I thank Ursula Kaempf, Marina Kuhn, Mark Dessing, Katerine Haft, Birgit Kugelberg, and Ho-Yan Mok for excellent technical help; Uwe Staerz, Lynn Ogata, John Kemp, and Fritz Melchers for antibodies; Hans Peter Stahlberger for excellent graphic work; Marie Kosco for morphological characterization of cell lines. I acknowledge the generous gifts of embryonic stem cells and kind help in establishing ES-cell technology by Rolf Kemler, Elizabeth Robertson, and Colin Stewart. I thank Steve Bauer for helpful discussions; Roland Lauster, Charley Steinberg, Steve Bauer, Richard Scheuermann, and Fritz Melchers for critical reading of the manuscript; and Nicole Schoepflin for preparing the manuscript. I am grateful to Fritz Melchers for introducing me to the field of developmental immunology and for his support and encouragement throughout this study.

The Basel Institute for Immunology was founded and is supported by F. Hoffman-La Roche and Co., Ltd., Basel, Switzerland.

*(Received April 12, 1991)*

*(Accepted August 7, 1991)*

### REFERENCES

Clynes R., Wax J., Stanton L.W., Smith-Gill S., Potter M., and Marcu K.B. (1988). Rapid induction of IgM-secreting murine plasmacytomas by pristane and an immunoglobulin heavy-chain promoter/enhancer-driven c-*myc*/v-Ha-*ras* retrovirus. Proc. Natl. Acad. Sci. USA **85:** 6067–6071.

Coffman R.L., and Weissman I.L. (1981). B220: A B cell-specific member of the T200 glycoprotein family. Nature **289:** 681–683.

Cudennec C.A., and Johnson G.R. (1981). Presence of multipotential hematopoietic cells in the teratocarcinoma cultures. J. Embryol. Exp. Morph. **61:** 51–59.

Cudennec C.A., and Nicolas J.-F. (1977). Blood formation in a clonal cell line of mouse teratocarcinoma. J. Embryol. Exp. Morph. **38:** 203–210.

DeChiara T.M., Efstratiadis A., and Robertson E.J. (1990). A growth-deficiency phenotype in heterozygous mice carrying an insulin-like growth factor II gene disrupted by targeting. Nature **345:** 78–80.

Dennert G., Hyman R., Lesley J., and Trowbridge I.S. (1980). Effects of cytotoxic monoclonal antibody specific for T200 glycoprotein on functional lymphoid cell populations. Cell. Immunol. **53:** 350–364.

Doetschman T., Eistetter H., Katz M., Schmidt W., and Kemler R. (1985). The in vitro development of blastocyst-derived embryonic stem cell lines: Formation of visceral yolk sac, blood islands and myocardium. J. Embryol. Exp. Morph. **87:** 27–45.

Doetschman T., Gregg R.G., Maeda N., Hooper M.L., Melton D.W., Thompson S., and Smithies O. (1987). Targetted correction of a mutant HPRT gene in mouse embryonic stem cells. Nature **330:** 576–578.

Goodman T., and LeFrancois L. (1989). Intraepithelial lymphocytes. Anatomical site, not T cell receptor form, dictates phenotype and function. J. Exp. Med. **170:** 1569–1587.

Gorman C.M., Rigby P.W.J., and Lane D.P. (1985). Negative regulation of viral enhancers in undifferential embryonic stem cells. Cell **42:** 519–526.

Gossler A., Doetschman T., Korn R., Serfling E., and Kemler R. (1986). Transgenesis by means of blastocyst-derived embryonic stem cell lines. Proc. Natl. Acad. Sci. USA **83:** 9065–9069.

Hardy R.R., Kemp J.D., and Hayakawa K. (1989). Analysis of lymphoid population in Scid mice; detection of a potential B lymphocyte progenitor population present at normal levels in Scid mice by three color flow cytometry with B220 and S7. Curr. Top. Microbiol. Immunol. **152:** 19–25.

Havran W.L., Poenie M., Kimura J., Tsien R., Weiss A., and Allison J.P. (1987). Expression and function of the CD3-antigen receptor on murine $CD4^+8^+$ thymocytes. Nature **330:** 170–173.

Karasuyama H., and Melchers F. (1988). Establishment of mouse cell lines which constitutively secrete large quantities of interleukin 2, 3, 4 or 5, using modified cDNA expression vectors. Eur. J. Immunol. **18:** 97–104.

Kincade P.W., Lee G., Watanabe T., Sun L., and Scheid M.P. (1981). Antigens displayed on murine B lymphocyte precursors. J. Immunol. **127:** 2262–2268.

Kohler G., Fischer-Lindahl K., and Heusser C. (1981). Characterization of a monoclonal anti-H-$2K^b$ antibody. *In:* The Immune system, vol. 2, Steinberg C., Lefkovits I., Eds. (Basel, Switzerland: Karger), pp. 202–208.

Koller B.H., Marrack P., Kappler J.W., and Smithies O. (1990). Normal development of mice deficient in $\beta_2$M, MHC class I proteins, and $CD8^+$ T cells. Science **248:** 1227–1230.

Koller B.H., and Smithies O. (1989). Inactivating the $\beta_2$-microglobulin locus in mouse embryonic stem cells by homologous recombination. Proc. Natl. Acad. Sci. USA **86:** 8932–8935.

Koo G., and Peppard J. (1984). Establishment of monoclonal anti-NK1.1 antibody. Hybridoma **3:** 301–303.

Kubo R.T., Born W., Kappler J.W., Marrack P., and Pigeon M. (1989). Characterization of a monoclonal antibody which detects all murine *αb* T cell receptors. J. Immunol. **142:** 2737–2743.

Labastie M.-C., Thiery J.-P., and Le Douarin N.M. (1984). Mouse yolk sac and intraembryonic tissues produce factors able to elicit differentiation of erythroid burst-forming units and colony-forming units, respectively. Proc. Natl. Acad. Sci. USA **81:** 1453–1456.

Ledbetter J.A., and Herzenberg L.A. (1979). Xenogeneic monoclonal antibodies to mouse lymphoid differentiation antigens. Immunol. Rev. **47:** 63–90.

Lindenbaum M.H., and Grosveld F.G. (1990). An in vitro globin gene switching model based on differentiated embryonic stem cells. Genes and Dev. **4:** 2075–2085.

Mansour S.L., Thomas K.R., and Capecchi M.R. (1988). Disruption of the proto-oncogene int-2 in mouse embryo-derived stem cells: A general strategy for targeting mutations to non-selectable genes. Nature **336:** 348–352.

Martin G.R. (1977). Teratocarcinoma and mammalian embryogenesis. *In:* Cell interactions in differentiation, Silver L.M., Martin G.R., and Strickland S., Eds. (New York: Academic Press), pp. 59–75.

Martin G.R., and Evans M.J. (1975). Differentiation of clonal lines of teratocarcinoma cells: Formation of embryoid bodies *in vitro*. Proc. Natl. Acad. Sci. USA **72:** 1441–1445.

Melchers F. (1977). B lymphocyte development in fetal liver. Eur. J. Immunol. **7:** 476–481.

Melchers F., and Abramczuk J. (1980). Murine embryonic blood between day 10 and 13 of gestation as a source of immature precursor B cells. Eur. J. Immunol. **10:** 763–767.

Mishell R.I., Bradley L.M., Chen Y.U., Grabstein K.H., Mishell B.B., Shiigi J.M., and Shiigi S.M. (1978). Inhibition of steroid-induced immune suppression by adjuvant stimulated accessory cells. J. Reticuloendothel. Soc. **23:** 439–447.

Mishell R.I., and Dutton R.W. (1967). Immunization of dissociated spleen cell cultures from normal mice. J. Exp. Med. **126:** 423–428.

Ogawa M., Nishikawa S., Ikuta K., Yamamura F., Naito M., Takahashi K., and Nishikawa S.-I. (1988). B cell ontogeny in murine embryo studied by a culture system with the monolayer of a stromal cell clone, ST2: B cell progenitor develops first in the embryonal body rather than in the yolk sac. EMBO J. **7:** 1337–1343.

Owen J.J.T., Raff M.C., and Cooper M.D. (1975). Studies on the generation of B lymphocytes in the mouse embryo. Eur. J. Immunol. **5:** 468–473.

Ozato K., Mayer N., and Sacks D. (1980). Hybridoma cell lines secreting monoclonal antibodies to mouse H-2 and Ia antigens. J. Immunol. **124:** 533–540.

Ozato K., Wan Y.-J., and Orrison B.M. (1985). Mouse major histocompatibility class I gene expression begins at midsomite stage and is inducible in earlier-stage embryos by interferon. Proc. Natl. Acad. Sci. USA **82:** 2427–2431.

Paige C.J., Kincade P.W., Moore M.A.S., and Lee G. (1979). The fate of fetal and adult B-cell progenitors grafted into immunodeficient CBA/N mice. J. Exp. Med. **150:** 548–563.

Righi M., Pierani A., Boglia A., De Libero G., Mori L., Marini V., and Ricciardi-Castagnoli P. (1989). Generation of new oncogenic murine retroviruses by cotransfection of clones AKR and MH2 proviruses. Oncogene **4:** 223–230.

Robertson E.J. (1987). Teratocarcinomas and embryonic stem cells, a practical approach, Robertson E.J., Ed. (Oxford, Washington, D.C.: IRL press).

Rolink A., Kudo A., Karasuyama H., Kikuchi Y., and Melchers F. (1991). Long-term proliferating early pre B cell lines and clones with the potential to develop to surface Ig-positive, mitogen reactive B cells *in vitro* and *in vivo*. EMBO J. **10:** 327–336.

Rosenberg N., and Baltimore D. (1976). A quantitative assay for transformation of bone marrow cells by Abelson murine leukemia virus. J. Exp. Med. **143:** 1453–1463.

Rosenberg N., Baltimore D., and Scher C.D. (1975). In vitro transformation of lymphoid cells by Abelson murine leukemia virus. Proc. Natl. Acad. Sci. USA **72:** 1932–1936.

Schmitt R.M., Bruyns E., and Snodgrass H.R. (1991). Hematopoietic development of embryonic stem cells in vitro: Cytokine and receptor gene expression. Genes & Devel. **5:** 728–740.

Schwartzberg P.L., Goff S.P., and Robertson E.J. (1989). Germ-line transmission of a c-*abl* mutation introduction by targeted gene disruption in ES cells. Science **246:** 799–803.

Shiigi S.M., and Mishell R.I. (1975). Sera and the in vitro induction of immune responses. I. Bacterial contamination and the generation of good fetal bovine sera. J. Immunol. **115:** 741–746.

Solter D., Knowles B.B. (1978). Monoclonal antibody defining a stage-specific mouse embryonic antigen (SSEA-1). Proc. Natl. Acad. Sci. USA **75:** 5565–5569.

Springer T., Galfré G., Secher D.S., and Milstein C. (1979). Mac-1: A macrophage differentiation antigen identified by monoclonal antibody. Eur. J. Immunol. **9:** 301–306.

Strasser A. (1988). PB76: A novel surface glycoprotein preferentially expressed on mouse pre-B cells and plasma cells detected by the monoclonal antibody G-5-2. Eur. J. Immunol. **18:** 1803–1810.

Thompson S., Clarke A.R., Pow A.M., Hooper M.L., and Melton D.W. (1989). Germ line transmission and expression of a corrected HPRT gene produced by gene targeting in embryonic stem cells. Cell **56:** 313–321.

Troppmair J., Potter M., Wan J., and Rapp U.R. (1989). An altered v-*raf* is required in addition to v-*myc* in J3V1 virus for acceleration of murine plasmacytomagenesis. Proc. Natl. Acad. Sci. USA **86:** 9941–9945.

von Boehmer H. (1990). Developmental biology of T cells in T cell-receptor transgenic mice. Annu. Rev. Immunol. **8:** 531–536.

Wilde D.B., Marrack P., Kappler J., Dialynas D.P., and Fitch F.W. (1983). Evidence implicating L3T4 in class II MHC antigen reactivity; monoclonal antibody GK1.5 (anti-L3T4a) blocks class II MHC antigen-specific proliferation, release of lymphokines, and binding by cloned murine helper T lymphocyte lines. J. Immunol. **131:** 2178–2183.

Wiles M.V., and Keller G. (1991). Multiple hematopoietic lineages develop from embryonic stem (ES) cells in culture Development **111:** 259–267.

Wu L., Scollay R., Egerton M., Pearse M., Spangrude G.J., and Shortman K. (1991). CD4 expressed on earliest T-lineage precursor cells in the adult murine thymus. Nature **349:** 71–73.

Zijlstra M., Li E., Sajjadi F., Subramani S., and Jaenish R. (1989). Germ-line transmission of a disrupted $\beta_2$-microglobulin gene produced by homologous recombination in embryonic stem cells. Nature **342:** 435–438.

